# Treatment of Kleine-Levin Syndrome With Intranasal Photobiomodulation and Methylene Blue

**DOI:** 10.7759/cureus.18596

**Published:** 2021-10-08

**Authors:** Michael Hamper, Paolo Cassano, Jay Lombard

**Affiliations:** 1 Neurology, Charles E. Schmidt College of Medicine, Florida Atlantic University, Boca Raton, USA; 2 Psychiatry, Massachusetts General Hospital, Boston, USA; 3 Neurosciences, Root Cause Medicine Practice, Tarrytown, USA

**Keywords:** i-pbm, methylene blue, red light, intranasal photobiomodulation, kleine-levin syndrome

## Abstract

Kleine-Levin syndrome (KLS) is a rare neuropsychiatric disorder, characterized by recurrent episodes of idiopathic hypersomnia, and cognitive and behavioral abnormalities, such as memory loss and child-like language. There is no definitive etiology for KLS; however, there are hypotheses of genetic predisposition, autoimmune mechanisms, and abnormal thalamic and hypothalamic functioning. Similarly, there is no definitive treatment for KLS as one method may be beneficial for one patient and not for another. We present a case of KLS in a patient who has no clinical improvement in symptoms with a variety of treatments. The parents of the patient agreed to attempt a trial of intranasal photobiomodulation (i-PBM) with red light, in combination with methylene blue (MB). The patient showed remission of the KLS episode following treatment with no further KLS episodes reported after treatment.

## Introduction

Kleine-Levin syndrome (KLS) is a rare neurological disorder characterized by recurrent episodes of hypersomnia, coupled with behavioral and cognitive abnormalities [1]. Cognitive abnormalities will include derealization and may involve memory loss and altered perception. Behavioral abnormalities may include apathy and child-like language [1]. Episodes may last several days to several weeks, or longer. Most patients are asymptomatic between episodes with full return to normal functioning; however, some patients may experience a mild cognitive deficiency in between episodes [2].

The causes and mechanisms of KLS are unknown; however, there are several hypotheses. The first is based on genetics. Gene-wide association studies have shown a link to variants in the TRANK1 gene loci, which is a major gene in bipolar disorder. It has been hypothesized that variants in the TRANK1 region may increase the risk of developing KLS, with a correlation between TRANK1 polymorphisms and difficult births, in patients who have KLS [3]. A second hypothesis is that there are autoimmune mechanisms that lead to the development of KLS, or at least, contribute to the symptoms. This hypothesis stems from the clinical observation that in most patients a bacterial or viral-like illness precedes (and appears to trigger) an episode with KLS symptoms [4,5]. Early studies revealed that some patients have an increase in the frequencies of HLA alleles, such as DQB1*0201 and HLA-DQB1*02 [6,7]. However, these findings have not been successfully replicated in large cohort studies. A number of patients with KLS had significantly higher levels of serum vascular cell adhesion molecule-1 (VCAM1) cytokine, within and in between episodes, compared to healthy controls, which may be indicative of an underlying inflammatory response [8]. Third, given that hypersomnolence is a cardinal symptom, it is suggested that KLS results from a dysfunction in the hypothalamus. The most consistent finding is hypoperfusion, demonstrated with functional MRI and/or single-photon emission computed tomography (SPECT) studies [9]. Of note, derealization - a key symptom of KLS - may result from decreased perfusion and metabolism in the thalamus, hypothalamus, and parieto-temporal junctions [9]. CT- and MRI-based imaging has consistently shown normal anatomy, which furthers support for a functional abnormality, such as hypoperfusion and hypometabolism, rather than a structural hypothesis [4].

The lack of a definitive etiology of KLS, coupled with the level of rarity, creates a challenge for diagnosis, which remains exclusively clinical. As a result, many prospective KLS patients take months, sometimes years, before obtaining an accurate diagnosis of the disorder. The current diagnostic criteria include both recurrent hypersomnia (RH) and KLS. RH criteria include: recurrent episodes of sleep lasting longer than two days, episodes have a frequency of at least once per year, cognitive functioning and behavior tend to be normal during the inter-episodic periods, and not caused by another medical or psychological disorder, medical use, or substance abuse. The diagnostic criteria for KLS include (in addition to RH): cognitive abnormalities, e.g., derealization, confusion, and memory loss, behavioral changes, e.g., uncharacteristic for the “normal” state of the patient, and apathy, hypersexuality, and hyperphagia [10]. It is important to note that - while hypersomnia, behavioral changes, and cognitive abnormalities define the symptomatic, episodes of KLS - hypersexuality and/or hyperphagia can be missing.

Unfortunately, there are no biomarkers for the diagnosis of KLS. Cases of KLS may resemble other diseases, hence the necessity of accurate differential diagnosis to ascertain the idiopathic nature of the syndrome. Given the acute onset of the episodic manifestations of KLS, common causes of mental status changes need to be ruled out before entertaining the suspicion of KLS. Patients with KLS may undergo a workup for acute confusion and substance abuse, and imaging to rule out a stroke or status epilepticus. Laboratory tests should include ammonia, folate, pyruvate, and lactate, evaluation for endocrinopathies, and for viral or bacterial infections. Encephalitis, head trauma, and other encephalopathies may mimic the symptoms of KLS; clinically, these differential diagnoses do not follow a remitting-relapsing disease course as seen in KLS. Relatedly, psychiatric disorders, such as depression, bipolar disorder, and conversion disorder need to be ruled out.

Treatment of KLS has previously been addressed symptomatically. Stimulants can be trialed to address hypersomnia [11]. However, this method fails to address cognitive and behavioral abnormalities and may result in irritability from treatment. A small cohort of patients has experienced benefit from clarithromycin, which appears to be an antagonist to these positive modulators of GABA-a receptors, as it is believed that some hypersomnolence disorders are associated with γ-aminobutyric acid (GABA)-a receptors [12]. Patients administered with the antibiotic showed that measures of sleepiness improved during treatment with clarithromycin [13]. However, this method has failed to address other characteristics of KLS and may not outweigh the long-term administration of an antibiotic. Lithium has shown great promise in patients treated with the medication. It has been found that Lithium may increase the duration of the inter-episodic period over time [3,14]. Anticonvulsants, particularly valproate, have been attempted with the suspicion of thalamic dysfunction and may be used as an alternative to lithium [15]. Some patients treated with methylprednisolone resulted in a beneficial response of shorter episode duration, which may be related to the potential autoimmune mechanisms involved [16]. Ultimately, the treatment of KLS is trial-and-error as one method may be beneficial to one patient, while it may not be beneficial to another. As described, and with most (if not all) pharmacological agents have limitations to their efficacy and therapeutic effects. The present study presents a novel noninvasive treatment with the potential to successfully treat KLS.

## Case presentation

A 15-year-old male presented with an acute episode of altered mental status. This episode of mental status changed began proceeding a flu-like illness, which resolved upon treatment with Tamiflu and minocycline. However, the psychosis continued after the resolution of the flu-like illness. The patient had no prior medical or psychiatric history. The patient became progressively lethargic and irritable. The symptoms further progressed to include hypersomnolence, apathy, derealization, and verbal aggressiveness. The patient refused to tend to his basic activities of daily living and eventually required hospitalization.

At admission, the patient was markedly lethargic, but afebrile and without meningeal signs, and general and neurological examinations were unremarkable during the episode. Magnetic resonance imaging (MRI) with gadolinium contrast did not show any structural abnormalities, space-occupying lesions, or abnormal enhancement. Electroencephalogram (EEG) testing was negative for epileptiform activity and for paroxysmal slowing. Serological tests for Lyme disease, HSV, EBV, IgG, and IgM were all negative.

This syndrome resolved within 10 days but was followed by recurrent episodes of longer duration - lasting two to three weeks - which included an obsession with food, depression, anxiety, paranoia, and social withdraw. Of note, some episodes were marked by hypomanic-like symptoms, such as the decreased need for sleep and uncharacteristically by dysmorphic body image. The patient was officially diagnosed with KLS, at 15 years of age, after being reevaluated at a tertiary care center, and after prolonged EEG, MRI, and CSF tests were repeated and confirmed as unrevealing. The patient continued to have recurrent episodes, commonly preceded by sinusitis.

Upon diagnosis, the patient began treatment with Lithium (600 mg in the AM and PM), which the patient remained continued for three years. Early in the disease course, the patient’s episodes were cyclical, occurring one to two times a month, with each episode lasting approximately two weeks. Over the course of three years, the patient’s episodes became progressively less frequent. At 18 years old, the patient’s episode frequency was approximately twice per year. Over the disease course, a variety of medications were attempted (in addition to lithium), including Modafinil, IV methylprednisolone, atypical neuroleptics, such as Seroquel and Amantadine. All treatment options resulted in no improvement, other than lithium, which may be the reason for less frequent episodes. Furthermore, the patient reported side effects while on the mentioned medications, including tingling and jitteriness in the extremities, and intestinal discomfort.

At 18-years-old, the patient experienced a flu-like illness, which “triggered” a prolonged episode of longer than the typical duration, extending close to 6 months. The family consented to the off-label use of intranasal photobiomodulation (i-PBM) with a red light at 633 nanometers (nm) (Vielight 633 Light Therapy), nightly for 25 minutes in conjunction with methylene blue (MB) at 10 milligrams (mg) orally, one hour prior to the use of i-PBM. Parameters/specifications for the i-PBM therapy device can be seen in Table 1. Within one week of treatment, the patient began to normalize with remission of his hypersomnia, and of his cognitive and behavioral abnormalities. After one month, the patient and family members noted the lack of recurrence of KLS episodes, as well as a return to socialization with friends and family. Six months after starting i-PBM plus MB, the patient enrolled in college. At 20 years old, two years after treatment with i-PBM and MB, the patient has not experienced a relapse of an episode, has been taking any other medications, nor has reported any adverse effects.

**Table 1:** Vielight 633 light therapy parameters Specifications of the Vielight treatment device used by the patient.

Parameter	Specification
Wavelength	633 nm
Power	8 mW
Peak Irradiance	7.6 mW/cm^2^
Pulsing	CW
Duty Cycle	100%
Fluence	11.4 J/cm^2^
Treatment Window	1 cm^2^
Time	25 min./nostril
Total Intranasal Energy	12 J (R. nostril 1x/day)

## Discussion

**Figure 1:**
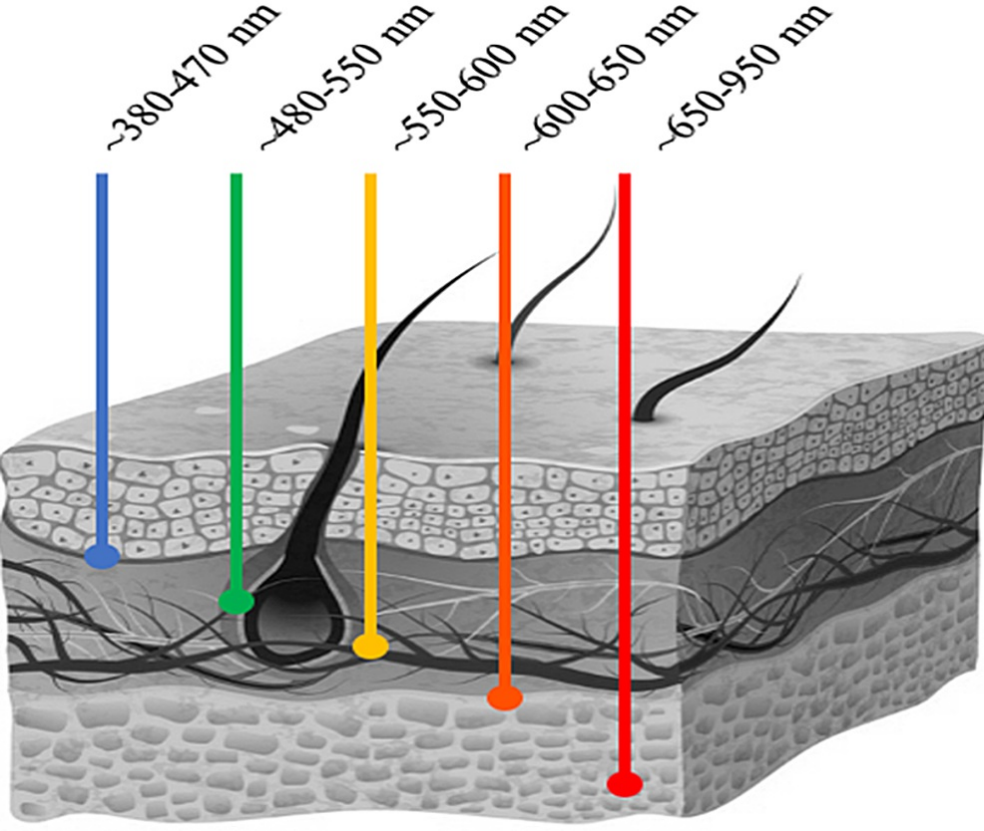
Depth of light penetration per wavelength Illustration depicting the penetrative depth through tissue based on the wavelength of light. Each color of light corresponds to a respective wavelength (in nanometers) and its associated color, e.g., the blue line is depicting the depth of blue light with its corresponding wavelength. Figure adapted from Avci et al. [24].

KLS poses a great challenge to treat as there is no definite etiology and many symptoms, both cognitive and behavioral. Fortunately, the patient in the present study accepted a novel experimental treatment of MB in conjunction with i-PBM. This treatment method was proposed as many patients with KLS have cerebral hypoperfusion and/or hypometabolism in particular areas of the brain. The use of red light for therapeutic reasons is called photobiomodulation (PBM). Red light has a short wavelength and high frequency to penetrate into the tissue enough to elicit changes in physiology (Figure 1), while not causing detrimental effects.

This PBM therapy may have modulated the brain activity during a resting and/or active state of the patient in the current study. It has been shown to evoke regions including the putamen, primary somatosensory, and parietal association cortex, with an overall effect of decreasing activity [17]. However, PBM therapy may affect the connectivity of the cortical region during a period of functional activity, i.e., when performing a task [17]. The modulation of neuronal activity is reported to be a result of manipulating membrane ion channels such as changing the influx of calcium ions [18]. The previous studies have found that wavelengths between 650 and 808 nm promote an elevation of intracellular Ca2+, suspected to be from an influx of intracellular Ca2+ and Ca2+ release from the endoplasmic reticulum with no effect observed at a greater wavelength [19]. Membrane potential can be sensitive to fluctuations in modifications in enzymic activities, such as phosphorylation of electron transport chain (ETC) enzymes, leading to the idea that mitochondrial reactive oxygen species (ROS) may play a role in cerebral perfusion [20]. In the rat model, studies have shown that treatment with PBM therapy led to a reduction of vascular infarction via inhibition of mitochondrial cytochrome c oxidase (COX), and thus, a decrease in the production of ROS [21]. A recent study investigated an immediate effect of i-PBM on blood flow, confirmed with SPECT imaging in patients with ischemic events - only a single session of i-PBM improved cerebral blood flow [22]. It is important to note that the effects of intranasal irradiation are systemic [23,24]. Anatomically, the olfactory bulb is a direct line to the brain, lying superior to the nasal cavity and inferior to the frontal lobe. Photons from the PBM therapy could directly reach the fibers of the olfactory nerve and the prefrontal cortex, which is connected to the thalamus and hypothalamus, critical areas in KLS [22]. Therefore, the effects on the brain are likely mediated by circulating mitochondria from direct irradiation of blood within the nasal capillaries via the anti-inflammatory and oxidative mechanisms described. Furthermore, it is reasonable to suspect that light can reach sensory terminations of the olfactory and indirectly affect the limbic system because the light cannot penetrate deeper at the power and wavelength used. The effects of the PBM therapy propose that modulation of mitochondrial enzyme activity may deflate the effect of hypoperfusion, a critical symptom in KLS.

MB, first synthesized as a textile dye, has shown great medicinal promise in applications beginning in the 1890s [25]. Recent work has shown the MB has a major effect on mitochondrial mechanisms as a catalytic redox recycler through promoting respiration via re-oxygenating leucomethylene blue by receiving electrons from NADH in complex I [26]. Oxidative damage commonly impairs complex IV, yet is bypassed by MB as it increases the activity of the complex [27]. Thus, MB has been shown to be neuroprotective with respect to its antioxidative capacity, which can be beneficial in an environment affected by ischemia or hypoperfusion commonly seen in KLS. Prior studying has shown that MB crosses the blood-brain barrier, preserving its integrity, and increasing circulation via inhibition of NOS, and decreasing lipid peroxidation and inflammation [28]. The idea that enhancing cognition with MB is not far stretched. As discussed, MB can improve mitochondrial function and decrease oxidative stress, which can improve neuron's effective function, therefore, improving basal neurophysiology and improve cognitive tasks. Human fMRI studies have shown that low-dose MB administration led to an increase in activity in the bilateral insular cortex during attention and memory tasks, as well as enhancing memory retrieval [29] Using MB and PBM in conjunction may promote a greater benefit than using one method over the other. As discussed, both can upregulate cellular respiration in the mitochondria with the benefits of neuroprotection from degradation. MB accomplishes increased cytochrome oxidase expression through supporting the ETC and PBM directly energizes the oxidase through photon absorption [30]. However, it is important to consider the hermetic response of MB as a high dose will elicit an opposite effect compared to a low dose, i.e., low doses of MB elicit responses that increase cytochrome oxidase activity, while high doses (beyond the hermetic/biphasic zone) decreases the cytochrome oxidase activity [31]. PBM has a similar relationship. At lower levels, i.e., less energy/time of fluency, induces an increase in membrane activity, while the effects decrease at higher fluences [32]. Thus, it is important for one to refer treatment with MB and PBM specifying the doses as high doses may cause adverse effects, but within lower parameters may lead to great benefit.

There are limitations to this study that should be acknowledged. This study contains one subject and is not a longitudinal study. KLS has been known to decrease in severity over time, and thus, it may be difficult to determine if the treatment is the exact cause for the dissolution of episodes. However, the gradual dissolution of episodes is more common in patients after many years. The patient presented in this study had only been experiencing episodes for three years prior to treatment with MB and i-PBM, and no medications have been used since the novel trial, with no report of a relapse post-treatment. Furthermore, the use of i-PBM is available for commercial purchase, MB can be obtained with the help of patients’ practitioners, and there have been no reported side effects - a great benefit to this modality, which improves accessibility and likelihood for more trials. We recommend that future studies include fMRI and PET imaging before and after treatment with MB and i-PBM. With all that has been discussed, i-PBM may improve cognition when used in conjunction with MB to successfully improve cognitive functioning in cases of hypoperfusion, like those in KLS, and ameliorate the major characteristic of derealization in the disorder as witnessed in the present study.

## Conclusions

KLS) is a rare neuropsychiatric disorder, characterized by recurrent episodes of idiopathic hypersomnia, and cognitive and behavioral abnormalities. There is no definitive treatment for KLS and many treatments attempted may have adverse effects. However, our findings support a successful treatment of KLS with the administration of MB and i-PBM therapy. This treatment assisted in relinquishing the patient of an episode and prevented the relapse of another episode. The present case provides evidence for research using MB and i-PBM, along with imaging methods and larger cohorts of patients with KLS.
